# The Value of MRI Findings Combined With Texture Analysis in the Differential Diagnosis of Primary Ovarian Granulosa Cell Tumors and Ovarian Thecoma–Fibrothecoma

**DOI:** 10.3389/fonc.2021.758036

**Published:** 2021-10-27

**Authors:** Nai-yu Li, Bin Shi, Yu-lan Chen, Pei-pei Wang, Chuan-bin Wang, Yao Chen, Ya-qiong Ge, Jiang-ning Dong, Chao Wei

**Affiliations:** ^1^ The First Affiliated Hospital of USTC, Division of Life Sciences and Medicine, University of Science and Technology of China, Hefei, China; ^2^ Department of the Healthcare, GE of China, Shanghai, China

**Keywords:** granulosa cell tumor, fibrothecoma, thecoma, sex cord stromal tumors, magnetic resonance imaging, texture analysis

## Abstract

**Objective:**

This study aims to explore the value of magnetic resonance imaging (MRI) and texture analysis (TA) in the differential diagnosis of ovarian granulosa cell tumors (OGCTs) and thecoma-fibrothecoma (OTCA–FTCA).

**Methods:**

The preoperative MRI data of 32 patients with OTCA–FTCA and 14 patients with OGCTs, confirmed by pathological examination between June 2013 and August 2020, were retrospectively analyzed. The texture data of three-dimensional MRI scans based on T2-weighted imaging and clinical and conventional MRI features were analyzed and compared between tumor types. The Mann–Whitney *U*-test, *χ* ^2^ test/Fisher exact test, and multivariate logistic regression analysis were used to identify differences between the OTCA–FTCA and OGCTs groups. A regression model was established by using binary logistic regression analysis, and receiver operating characteristic curve analysis was carried out to evaluate diagnostic efficiency.

**Results:**

A multivariate analysis of the imaging-based features combined with TA revealed that intratumoral hemorrhage (OR = 0.037), log-sigma-20mm-3D_glszm_SmallAreaEmphasis (OR = 4.40), and log-sigma-2-0mm-3D_glszm_SmallAreaHighGrayLevelEmphasis (OR = 1.034) were independent features for discriminating between OGCTs and OTCA–FTCA (*P* < 0.05). An imaging-based diagnosis model, TA-based model, and combination model were established. The areas under the curve of the three models in predicting OGCTs and OTCA–FTCA were 0.935, 0.944, and 0.969, respectively; the sensitivities were 93.75, 93.75, and 96.87%, respectively; and the specificities were 85.71, 92.86, and 92.86%, respectively. The DeLong test indicated that the combination model had the highest predictive efficiency (*P* < 0.05), with no significant difference among the three models in differentiating between OGCTs and OTCA–FTCA (*P* > 0.05).

**Conclusions:**

Compared with OTCA–FTCA, intratumoral hemorrhage may be characteristic MR imaging features with OGCTs. Texture features can reflect the microheterogeneity of OGCTs and OTCA–FTCA. MRI signs and texture features can help differentiate between OGCTs and OTCA–FTCA and provide a more comprehensive and accurate basis for clinical treatment.

## Introduction

Ovarian sex cord stromal tumors are rare tumors that account for approximately 7% of all ovarian tumors. According to the 2014 World Health Organization (WHO) ovarian tumor histological classification, these tumors are divided into pure stromal tumors, pure sex cord tumors, luteinized thecoma associated with sclerosing peritonitis, and mixed sex cord stromal tumors. Pure stromal tumors include three subtypes: fibroma, cellular fibroma, and thecoma; these are mainly distinguished based on whether they comprise theca cells, lutein cells, fibroblasts, and fibrocytes. This group of tumors has overlapping features in multidirectional differentiation through histology, which makes it difficult to obtain a pathological diagnosis in some cases. Therefore, these tumors are traditionally named ovarian thecoma-fibrothecoma (OTCA–FTCA) (1, 2).

OTCA–FTCA and ovarian granulosa cell tumors (OGCTs) are the most common sex cord stromal tumors and have a low incidence relative to other ovarian tumors. These tumors are usually discovered by chance during gynecological examinations or routine physical examinations as the symptoms are nonspecific. OTCA–FTCA accounts for 0.5–1.0% of ovarian tumors, is generally benign, and has an excellent prognosis after resection, but a small proportion of these tumors (1.0–5.0%) are malignant (3, 4). OGCTs are rare sex cord stromal tumors with a low malignant potential and account for only 5% of all malignant ovarian tumors, with adult and juvenile forms of subtypes. OGCTs have a low degree of malignancy, show growth patterns of benign tumors, and have potentially malignant behaviors, including local invasion, recurrence, and metastasis (5, 6). OTCA–FTCA is mainly found in menopausal women, and less than 10% occur before age 30 (7, 8); however, OGCTs are more common in postmenopausal women, and the juvenile type is rare and typically occurs before 30 years of age. Sometimes these tumors share similar clinical manifestations (such as elevated estrogen levels leading to endometrial hyperplasia and irregular vaginal bleeding). These tumors can have similar imaging findings, such as combined with cystic degeneration, edema, and hemorrhage, which may cause misdiagnosis in radiography and inappropriate choice of treatment of clinicians (9–11). Therefore, the preoperative diagnosis of OTCA–FTCA and OGCTs is particularly important.

Magnetic resonance imaging (MRI) has high resolution in soft tissues that clearly reveal the lesion characteristics, relationship between the tumor and surrounding tissues, and the status of lymph node disease (9). In particular, the semiquantitative parameters deprived from diffusion-weighted imaging (DWI) have gradually become one of the important tools for evaluating ovarian tumors (12). Texture analysis (TA) has been widely adopted in the differential diagnosis of tumors in recent years and is considered to be an effective means to assess tumor heterogeneity. Not only MRI-based texture analysis but also CT texture-based analysis of the whole tumor has demonstrated high sensitivity and specificity for the characterization of ovarian tumors and may assist in characterizing the differences in ovarian tumor patients. The application of MRI-based texture features combined with conventional MRI features may assist in improving the differentiation of ovarian tumors. These findings, in turn, may guide diagnostic protocols for future patients and can help radiologists make appropriate follow-up decisions (3, 4, 7).

Therefore, the purpose of this study was to identify the best features for distinguishing between OTCA–FTCA and OGCTs through conventional MRI, TA, and the combination of the two diagnostic methods to improve the accuracy of preoperative imaging-based diagnoses and help clinicians choose appropriate treatment methods.

## Materials and Methods

### Clinical Information

This retrospective study was approved by the institutional review board of The First Affiliated Hospital of University of Science and Technology of China (USTC), and the requirement of written informed consent was waived. Between June 2013 and August 2020, 1,586 patients with clinically suspected adnexal disease who underwent 3.0-T MR examinations were reviewed through the picture archiving and communication system at the First Affiliated Hospital of the USTC. A total of 46 patients with histologically proven OGCTs (*n* = 14, 15–71 years of age) and OTCA–FTCA (*n* = 32, 24–94 years of age) were included in this study. The inclusion criteria were as follows: (1) surgically diagnosed tumor with a known pathological type (according to the 2014 WHO classification of ovarian tumors), (2) no intervention before the MRI examination, (3) lesion that could be measured and segmented on MRI, and (4) signed informed consent form provided before the examination.

### MRI Examination

MRI was performed using a 3.0-T system (Signa HDxT, GE Healthcare) with an eight-channel phased array coil. The routine MRI protocols used to assess the pelvic masses included axial T1-weighted imaging (T1WI), axial/sagittal T2-weighted imaging (T2WI), axial fat-suppressed T2WI (FS T2WI), DWI (*b* value = 0, 1,000 s/mm^2^), and multiple phases of contrast-enhanced (LAVA-FLEX) MRI. For the axial images, the transverse plane was perpendicular to the long axis of the uterine body; for the sagittal images, the longitudinal plane was parallel to the main body of the uterus. If contraindications were excluded, the patients were often given an intramuscular injection of 20 mg scopolamine 15 min before the examination to suppress MRI motion artefacts caused by peristalsis. Contrast-enhanced pelvic imaging was acquired at the arterial, venous, and delayed phases of contrast medium enhancement in axial planes, which were acquired at 25, 60, and 120 s after the intravenous injection of 0.1 mmol/kg gadodiamide (Omniscan, GE Healthcare) using an Ulrich power injector. Some of the scanning sequences and parameters are shown in **Table 1**.

**Table 1:** Partial list of MRI parameters.

SEQUENCE	TE (ms)	TR (ms)	Freq × phase	Nex	FOV	Slice thickness	Interval	Flip angle
FS T2WI	72.5	5,000	320 × 256	2	24 × 24	6	2	90°
T2WI	72.5	4,600	320 × 256	2	24 × 24	6	2	90°
Osag T2WI	72	4,500	320 × 320	2	28 × 28	4	1	90°
T1WI	7.5	500	352 × 192	2	32 × 32	6	2	90°
DWI (*b* = 0, 1,000 s/mm^2^)	/	5,000	96 × 130	6	32 × 32	6	2	90°
Oax LAVA-FLEX	1.4	5.8	320 × 224	1	34 × 31	4	0	15°
Osag LAVA-FLEX	1.3	6.8	268 × 224	1	28 × 25	4	0	15°

### Radiological Evaluation

Two radiologists (YuC and BS, with 10 and 7 years of experience in gynecological imaging, respectively) who were blinded to the histological results independently analyzed the MRI data of each participant, and discrepancies were resolved by consensus. The following MRI features were recorded and analyzed for the two groups: size (the maximum diameter of the tumor and the shortest perpendicular diameter measured on T2WI, the maximum upper and lower diameter of the tumor measured on sagittal T2WI, and the average size of the aboved diameters), endometrial hyperplasia (endometrium thickness greater than 5 mm after menopause and greater than 16 mm in premenopausal women) (12), apparent diffusion coefficient (ADC) value (10^3^ mm^2^/s) [mean value obtained from three measurements of a region of interest (ROI) manually placed in the solid components of the tumors and myometrium, and the calculated ratio; the ROI was drawn using GE AW4.5 workstation Functool-MADC software, and attempts were made to avoid tumor necrosis and cystic areas], enhancement degree, T2WI signal, and DWI signal of the solid component of the tumors (hypointense, isointense, or hyperintense compared with the myometrium at the same level), location (left or right), degree of cystic components (graded as 0–4°; grade 0 = no cystic change; grade 1 = area with cystic changes was ≤25%; 25% < grade 2 ≤ 50%; 50% < grade 3 ≤ 75%; and 75% < grade 4), cystic form (no cyst, mainly small sac, mainly large and mixed; small sac ≤1.0 cm, large sac >1.0 cm, or a mix of both), intratumoral hemorrhage (present or absent), and age (years, mean ± standard deviation, SD).

### Texture Feature Extraction

The images of OTCA–FTCA and OGCTs were manually segmented, and volumes were extracted using *ITK* Snap software (3.8.0, http://www.itksnap.org). ROIs were delineated around the tumor boundary for each section by two radiologists (YuC and BS). After tumor segmentation, AK software (Analysis Kit Version: 3.2.0; GE Healthcare) was used for texture feature extraction, and 1,316 features, such as the mean, entropy, energy, skewness, kurtosis, and standard deviation, were obtained in this study.

### Statistical Analysis

Continuous and categorical variables were compared using the *t*-test and *χ* ^2^/Fisher’s exact test, respectively. Continuous variables are expressed as the mean ± standard deviation, and categorical variables are expressed as the frequency and percentage (%). Continuous variables were first tested for normality to understand the data distribution, and the variables were tested as follows: (1) an independent-sample *t*-test was used to compare variables both conforming to a normal distribution, and (2) the Mann–Whitney *U*-test was used to compare variables conforming to a skewed distribution and variables conforming to a skewed distribution with those conforming to a normal distribution. Continuous and categorical variables showing significant differences were analyzed by multivariate logistic regression analysis with the forward step method to screen for independent discriminant features, which were used to construct the discriminating model. Receiver operating characteristic (ROC) curve and area under the curve (AUC) analyses were performed with MedCalc (version 19.5.3, https://www.medcalc.org/) to determine the overall diagnostic performance of the radiographic model, texture model, and combined model. SPSS 26.0 software (version 20.0, IBM, Armonk, NY, USA) was used for statistical analysis, and *P <*0.05 was considered statistically significant. The intraclass correlation coefficient (ICC) was used to evaluate the consistency between evaluator 1 and evaluator 2, and an ICC between 0.81 and 1.00 indicated good agreement.

## Results

### Pathological, Clinical, and Imaging Findings

**Figure 1:**
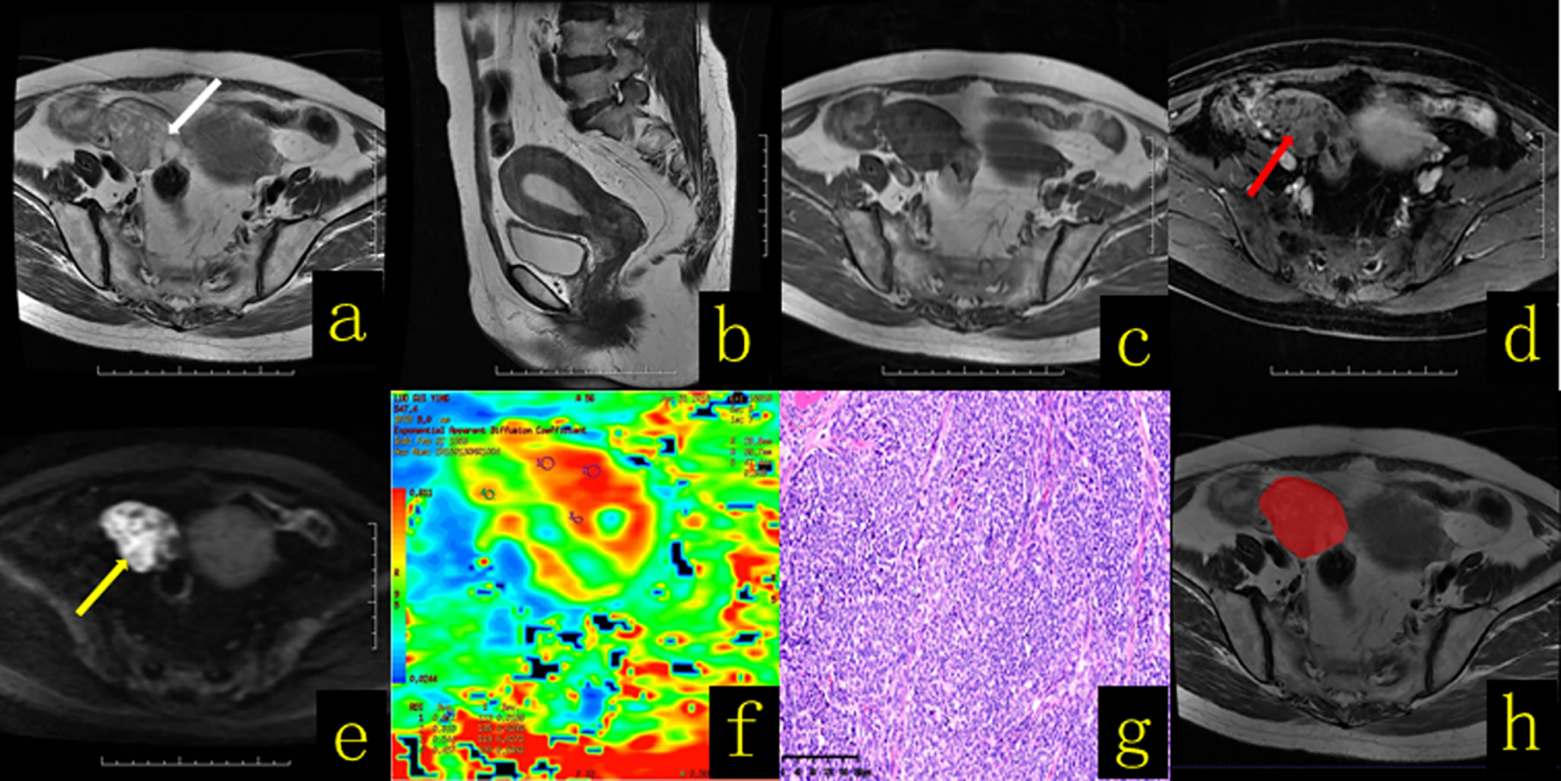
A 61-year-old female patient with an ovarian granulosa cell tumor. **(A)** Axial T2WI revealed a cystic solid mass in the right adnexal region that manifested with a “spongy” or “honeycomb” change (white arrow). **(B)** Sagittal T2WI showed thickening of the endometrium to a thickness of approximately 1.9 cm. **(C)** Axial T1WI revealed a cystic solid mass with a hypo–isointense signal. **(D)** On contrast-enhanced fat-suppressed T1WI, the solid components (red arrow) of the lesion showed mild and moderate enhancement, with a region resembling the myometrium. **(E)** On DWI-MRI (*b* = 1,000 s/mm^2^), the solid part of the lesion appeared hyperintense (yellow arrow), and the cystic part appeared hypointense. **(F)** The apparent diffusion coefficient (ADC) map showed that the average ADC value of the diffuse high-signal area was approximately 0.7 × 10^-3^ mm^2^/s. **(G)** Hematoxylin and eosin (H&E) staining (×100) showed that the tumor cells appeared as large islands, diffusely distributed in nests and rich in interstitial separation and blood vessels. **(H)** The texture analysis target area was delineated throughout the whole tumor layer by layer.

**Figure 2:**
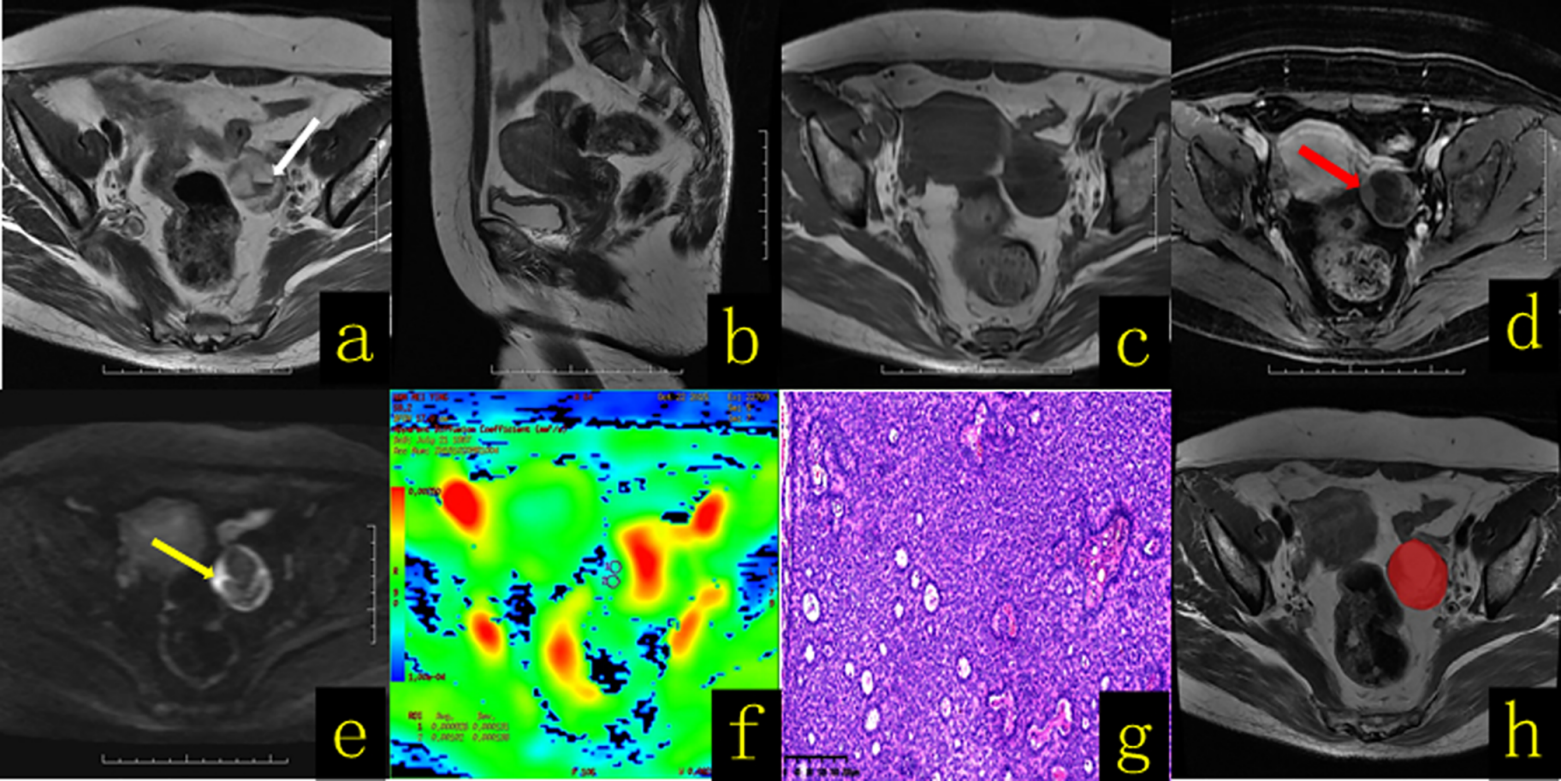
A 58-year-old female patient with an ovarian granulosa cell tumor. **(A)** Axial T2WI revealed a well-defined cystic solid mass in the left adnexal region, with fluid–fluid levels (hemorrhagic content, white arrow). **(B)** Sagittal T2WI showed no thickening of the endometrium. **(C)** Axial T1WI revealed a cystic solid mass with a hypo–isointense signal. **(D)** On contrast-enhanced fat-suppressed T1WI, the solid components (red arrow) of the lesion showed mild enhancement. **(E)** On DWI-MRI (*b* = 1,000 s/mm^2^), the solid part of the lesion (yellow arrow) appeared hyperintense. **(F)** The apparent diffusion coefficient (ADC) map showed that the average ADC value of the diffuse high-signal area was approximately 1.1 × 10^-3^ mm^2^/s. **(G)** Hematoxylin and eosin (H&E) staining (×100) showed that the tumor cells were solid tubular structures, and the tubules were composed of uniform cells containing Call–Exner bodies. **(H)** The texture analysis target area was delineated throughout the whole tumor layer by layer.

**Figure 3:**
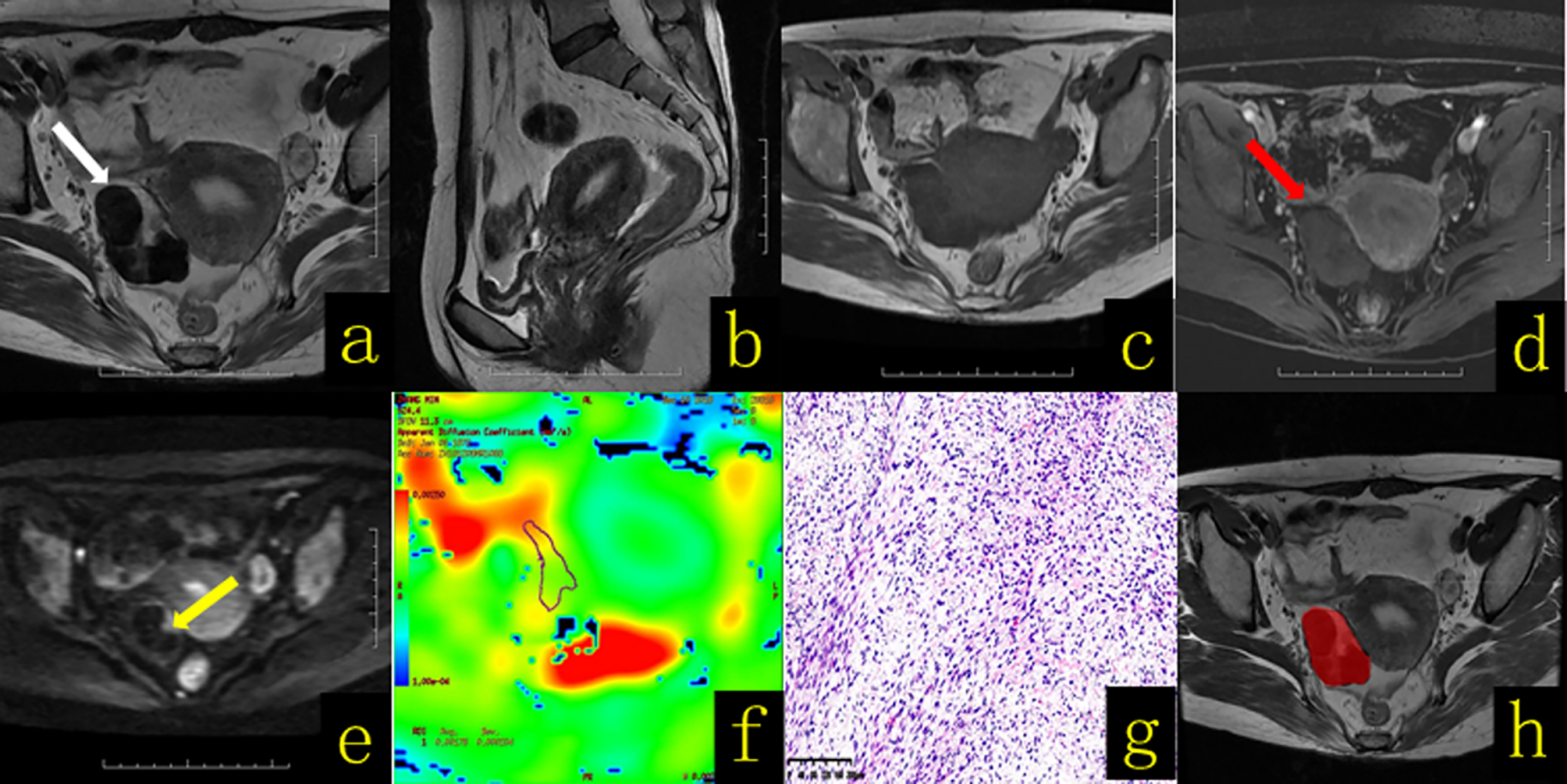
A 65-year-old female patient with right ovarian thecoma–fibrothecoma. **(A)** Axial T2WI revealed a solid mass in the right adnexal region (white arrow), showing mainly a low-signal mass with a semiarc shape and high signal at the left front edge. **(B)** Sagittal T2WI showed thickening of the endometrium to a thickness of approximately 1.2 cm. **(C)** Axial T1WI revealed a solid mass with hypo–isointense signal (white arrow). **(D)** On contrast-enhanced fat-suppressed T1WI, the solid components (red arrow) of the lesion showed mild enhancement. **(E)** On DWI-MRI (*b* = 1,000 s/mm^2^), the solid part of the lesion of the left front edge appeared hyperintense (yellow arrow). **(F)** The apparent diffusion coefficient (ADC) map showed that the average ADC value of the diffuse high-signal area was approximately 1.78 × 10^-3^ mm^2^/s. **(G)** Hematoxylin and eosin (H&E) staining (×100) showed that the tumor was composed of spindle cells and collagen fibers arranged in a mat-like pattern with interwoven bundles, and hyaline degeneration of fibrous tissue bands and intercellular edema were observed to varying degrees. The tumor cell nucleus was fusiform to oval, with sparse cytoplasm and containing a small amount of lipids; the mitotic index was <3/10 HPF. **(H)** The texture analysis target area was delineated throughout the whole tumor layer by layer.

The pathological diagnoses of all OTCA–FTCA and OGCTs were made by a pathologist (YuC, with 8 years of experience in gynecological tumors) according to the 2014 WHO ovarian sex cord stromal tumor histological classification. Finally, a total of 32 patients with OTCA–FTCA (mean age, 52.93 ± 12.39 years) and 14 patients with OGCTs (mean age, 49.93 ± 19.19 years) were enrolled in this study. The 14 patients with OGCTs included 12 adult and two juvenile patients, with eight patients with tumors in the right ovary, six patients with tumors in the left ovary, and three patients with endometrial hyperplasia (21%). There were 11 patients with cystic–solid changes (two small sacs, one large sac, and eight mixed types) (**Figure 1**) and 11 patients with hemorrhage signal (76%) in the tumor (**Figure 2**); the enhancement degree of the solid components of the tumor was mainly mild to moderate (eight patients with mild enhancement and six patients with moderate enhancement), with no patients with marked enhancement. Among the 32 patients with OTCA–FTCA, 23 patients had tumors in the right ovary, nine patients had tumors in the left ovary, and three patients had endometrial hyperplasia (9%); 18 solid masses (**Figure 3**) were observed, with 14 showing mainly cystic–solid changes (one small cyst, 10 large cysts, and three mixed). There were five patients (16%) with hemorrhage signals in the tumor. As shown in **Table 2**, the following four MRI-based characteristics were significantly different between the OGCTs and OTCA–FTCA groups: (1) the mean ADC value of the solid component (*z* = -1.982, *P* = 0.047), (2) the degree of enhancement of the solid component (*χ* ^2^/9.084, *P* = 0.003), (3) the cystic form (Fisher/0.006, *P* = 0.008), and (4) the presence of intratumoral hemorrhage (Fisher/0.000, *P*=0.000).

**Table 2:** Details of the clinical and MR imaging-based characteristics of 14 histologically proven OGCTs and OTCA–FTCA in 32 patients.

Characteristics	Category	OGCTs (*n* = 14)	OTCA–FTCA (*n* = 32)	^2^/Fisher/*z* value	*P*-value
Age (years)		49.93 ± 19.19	52.93 ± 12.39	z/-0.478	0.632
Size (maximum)	/	6.65 ± 4.60	8.08 ± 5.33	z/-0.967	0.333
Size (average)	/	6.47 ± 4.74	7.96 ± 5.18	z/-1.146	0.252
Mean ADC (10^3^ s/mm^2^)	/	1.27 ± 0.37	1.50 ± 0.32	z/-1.982	0.047
ADC (10^3^ s/mm^2^, ratio)	/	0.93 ± 0.24	1.05 ± 0.27	z/-1.695	0.090
Menopause	Postmenopausal	10 (71%)	21 (66%)	*χ* ^2^/0.149	0.699
	Premenopausal	4 (29%)	11 (34%)		
Endometrial hyperplasia	Present	3 (21%)	3 (9%)	Fisher/0.350	0.264
	Absent	11 (79%)	29 (91%)		
T2WI intensity (solid)	Hypointense	2 (14%)	12 (38%)	Fisher/0.102	0.084
	Isointense	6 (43%)	11 (34%)		
	Hyperintense	5 (36%)	3 (9%)		
	Mixed signal	1 (7%)	6 (19%)		
Location	Right	8 (57%)	23 (72%)	Fisher/0.495	0.327
	Left	6 (43%)	9 (28%)		
DWI intensity (solid)	Isointense	1 (7%)	5 (16%)	Fisher/0.175	0.149
	Hyperintense	1 (7%)	9 (28%)		
	Mixed	12 (86%)	18 (56%)		
Enhancement degree (solid)	Mild	8 (57%)	30 (94%)	*χ* ^2^/9.084	0.003
	Moderate	6 (43%)	2 (6%)		
	Marked	0 (0%)	0 (0%)		
Degree of cystic components	None	3 (21%)	18 (56%)	Fisher/0.149	0.229
	<25%	4 (29%)	4 (13%)		
	25–50%	1 (7%)	3 (9%)		
	50%~75%	1 (7%)	1 (3%)		
	>75%	5 (36%)	6 (19%)		
Cystic form	Small cyst	2 (14%)	1 (3%)	Fisher/0.006	0.008
	Large cyst	1 (7%)	10 (31%)		
	Mixed	8 (57%)	3 (9%)		
Intratumoral hemorrhage	Present	11 (76%)	5 (16%)	Fisher/0.000	0.000
	Absent	3 (24%)	27 (84%)		

### Diagnostic Performance of the Texture Features

Least absolute shrinkage and selection operator (Lasso) regression was performed in R (3.6.1, http://www.r-probject.org) to reduce the dimensionality of the features and filter and remove redundancy parameters (|*r*| > 0.8) to reduce the impact of data overfitting. First, the Mann–Whitney *U*-test was applied to the features to explore whether the features were significantly different between the two groups, and 123 features with *p <*0.05 were retained. Second, univariate logistic regression was applied to explore whether the features were discriminative between the two groups, and 78 features with *p <*0.05 were retained. Third, minimum redundancy and maximum correlation were applied to eliminate the redundant features and retain the features that were highly correlated with the label, and 10 features were retained. Then, the retained features were enrolled in backward stepwise multivariate logistic regression, and the final model was constructed. The explanation of the texture analysis features is shown in **Table 3**.

**Table 3:** Explanation of the texture analysis features.

Image type	Features	Feature explanation
log-sigma-2-0-mm-3D	glszm_SmallAreaEmphasis	Small area emphasis (SAE): SAE is a measure of the distribution of small size zones, with a greater value indicative of much smaller size zones and more fine textures
	$\frac{\Sigma_{i=1}^{N_{g}}\Sigma_{j=1}^{N_{s}}\frac{p(i,j)}{j^{2}}}{N_{z}}$	
	glszm_SizeZoneNonUniformityNormalized	SZNN measures the variability of size zone volumes throughout the image, with a lower value indicating more homogeneity among zone size volumes in the image. This is the normalized version of the SZN formula
	$\frac{\Sigma_{j=1}^{N_{s}}(\Sigma_{i=1}^{N_{g}}p{\left(i,j\right)}^{2})}{N_{z}}$	
	glszm_SmallAreaHighGrayLevelEmphasis	SAHGLE measures the proportion in the image of the joint distribution of smaller size zones with higher gray-level values
	$\frac{\Sigma_{i=1}^{N_{g}}\Sigma_{j=1}^{N_{s}}\frac{p(i,j)i^{2}}{j^{2}}}{N_{z}}$	
log-sigma-3-0-mm-3D	glcm_InverseVariance	Reflects the local variation of the image texture; so, if more uniformity was found in the different regions of the image texture, this indicates that the change is slower, the value will be larger, and vice versa
	$\sum_{k=1}^{N_{g}-1}\frac{p_{x-y}(k)}{k^{2}}$	
wavelet-LLH	glcm_MCC	Maximal correlation coefficient (MCC). The maximal correlation coefficient is a measure of complexity of the texture and 0 ≤ MCC ≤ 1. In case of a flat region, each GLCM matrix has shape (1, 1), resulting in just 1 eigenvalue.
	$\sqrt{\sum_{k=0}^{N_{g}}\frac{p(i,k)p(j,k)}{p_{x}(i)p_{y}(k)}}$	
wavelet-HLH_	glszm_SmallAreaHighGrayLevelEmphasis	Measures the proportion in the image of the joint distribution of smaller size zones with higher gray-level values
	$\frac{\Sigma_{i=1}^{N_{g}}\Sigma_{j=1}^{N_{s}}\frac{p(i,j)i^{2}}{j^{2}}}{N_{z}}$	
wavelet-HLL	glszm_LowGrayLevelZoneEmphasis	Measures the distribution of lower gray-level size zones, with a higher value indicating a greater proportion of lower gray-level values and size zones in the image
	$\frac{\Sigma_{i=1}^{N_{g}}\Sigma_{j=1}^{N_{s}}\frac{p(i,j)}{i^{2}}}{N_{z}}$	
lbp-3D-k	glszm_ZonePercentage	Measures the coarseness of the texture by taking the ratio of the number of zones and number of voxels in the region of interest (ROI). Values are in the range 1Np ≤ ZP ≤ 1, with higher values indicating a larger portion of the ROI consisting of small zones (indicates a finer texture)
	$\frac{N_{z}}{N_{p}}$	
lbp-3D-k	firstorder_Kurtosis	Kurtosis is a measure of the “peakedness” of the distribution of values in the image region of interest. A higher kurtosis implies that the mass of the distribution is concentrated towards the tail(s) rather than towards the mean. A lower kurtosis implies the reverse: that the mass of the distribution is concentrated towards a spike near the mean value
	$\frac{\mu_{4}}{\sigma^{4}}=\frac{\frac{1}{N_{p}}\Sigma_{i=1}^{N_{p}}{\left(X(i)-\bar{X}\right)}^{4}}{{\left(\frac{1}{N_{p}}\Sigma_{i=1}^{N_{p}}{\left(X(i)-\bar{X}\right)}^{2}\right)}^{2}}$	
original_shape_Sphericity	Sphericity	Sphericity is a measure of the roundness of the shape of the tumor region relative to a sphere. It is a dimensionless measure, independent of scale and orientation. The value range is 0 < sphericity ≤ 10 <sphericity ≤ 1, where a value of 1 indicates a perfect sphere (a sphere has the smallest possible surface area for a given volume, compared to other solids)
	$\frac{\sqrt[{3}]{36\pi V^{2}}}{A}$	

Reference: https://pyradiomics.readthedocs.io/en/latest/features.html.

The ICC was used to evaluate the consistency between radiologist 1 and radiologist 2 and was 0.81–1.00 (*P* < 0.001), indicating good consistency. Finally, the average of the two sets of data was used as the new texture data for statistical analysis. As shown in **Table 4**, the following six texture features were significantly different between the OGCTs and OTCA–FTCA groups: (1) log-sigma-2-0-mm-3D_glszm_SmallAreaEmphasis (SAE) (*z* = -4.201, *P* = 0.000), (2) log-sigma-2-0-mm-3D_glszm_SmallAreaHighGrayLevelEmphasis (*z* = -3.187, *P* = 0.340), (3) log-sigma-3-0-mm-3D_glcm_InverseVariance (*z* = -3.342, *P* = 0.001), (4) wavelet-LLH_glcm_MCC (*z* = -4.106, *P* = 0.001), (5) wavelet-HLH_glszm_SmallAreaHighGrayLevelEmphasis (*z* = -2.984, *P* = 0.003), and (6) wavelet-HLL_glszm_LowGrayLevelZoneEmphasis (*z* = -3.103, *P* = 0.002).

**Table 4:** Results of the univariate analysis of texture features that were significantly different between the OGCTs and OTCA–FTCA groups.

Features	OGCTs	OTCA–FTCA	Mann–Whitney *U*	*Z*-value	*P*-value
log-sigma-2-0-mm-3D_glszm_SmallAreaEmphasis	0.38 ± 0.094	0.70 ± 0.26	50.000	-4.201	0.000
log-sigma-2-0-mm-3D_glszm_SizeZoneNonUniformityNormalized	0.16 ± 0.059	397.89 ± 676.32	184.000	-0.955	0.340
log-sigma-2-0-mm-3D_glszm_SmallAreaHighGrayLevelEmphasis	92.85 ± 87.99	39.93 ± 73.47	92.000	-3.187	0.001
log-sigma-3-0-mm-3D_glcm_InverseVariance	0.33 ± 0.053	1.55 ± 1.35	84.000	-3.342	0.001
wavelet-LLH_glcm_MCC	0.64 ± 0.12	604.17 ± 873.98	52.000	-4.106	0.000
wavelet-HLH_glszm_SmallAreaHighGrayLevelEmphasis	52.32 ± 29.84	56.13 ± 203.67	99.000	-2.984	0.003
wavelet-HLL_glszm_LowGrayLevelZoneEmphasis	0.04 ± 0.06	3.93 ± 6.73	94.000	-3.103	0.002
lbp-3D-k_glszm_ZonePercentage	0.009 ± 0.003	99.68 ± 195.12	202.000	-0.525	0.599
lbp-3D-k_firstorder_Kurtosis	8.73 ± 4.06	99.65 ± 163.28	206.000	-0.430	0.667
original_shape_Sphericity	0.75 ± 0.04	24.14 ± 34.74	212.000	-0.286	0.775

### Diagnostic Performance of the Predictive Models Based on MRI Characteristics, Texture Features, and Combined Features

**Figure 4:**
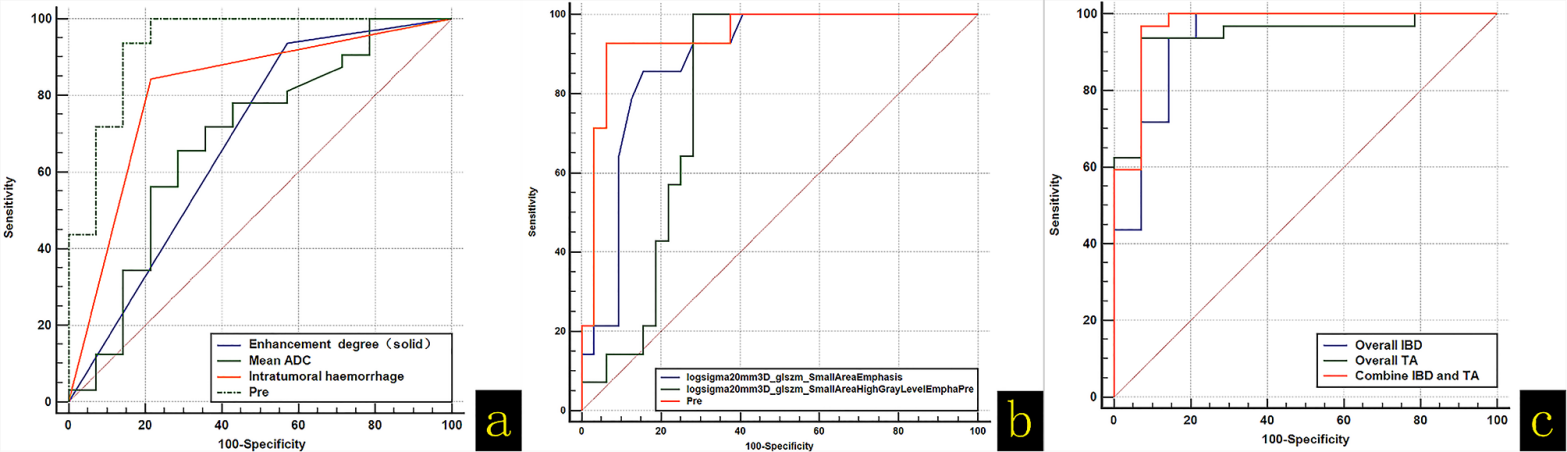
**(A)** (ROC) curve analysis of the diagnostic abilities of apparent diffusion coefficient values (average), enhancement degree, presence of intratumoral hemorrhage, and the prediction models. **(B)** ROC curve analysis of Log-sigma-2-0mm-3D_glszm_SmallAreaEmphasis, Log-sigma-2-0mm-3D_glszm_SmallAreaHighGrayLevelEmphasis, and the prediction models. **(C)** ROC curve analysis of the overall imaging-based diagnosis (IBD), overall texture analysis (TA), and combined IBD with TA models.

**Figure 5:**
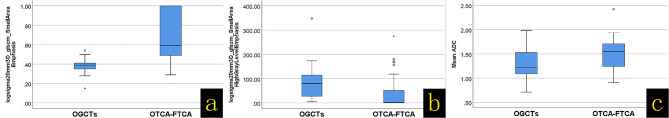
**(A)** Box-and-whisker plot of the Log-sigma-2-0mm 3D_glszm_SmallAreaEmphasis difference between OGCTs and OTCA–FTCA. **(B)** Box-and-whisker plot of the Log-sigma-2-0mm 3D_glszm_SmallAreaHighGrayLevelEmphasis difference between OGCTs and OTCA–FTCA. **(C)** Box-and-whisker plot of the Mean ADC difference between OGCTs and OTCA–FTCA.

The variables with significant differences in the univariate analysis were included in the multivariate logistic regression analysis for screening. As shown in **Table 5**, the overall imaging-based diagnosis (IBD) and overall TA prediction models based on MRI characteristics and texture features were established, respectively: (Y-IBD) = -10.04 + 6.67 × ADC (average) + 4.67 × enhancement degree (solid) (mild = 0, moderate = 1, marked = 3) - 4.63 × intratumoral hemorrhage (present = 0, absent = 1), and (Y-TA) = -11.39 + 33.18 × log-sigma-2-3D_glszm_SmallAreaEmphasis (x ± s) - 0.03 × log- sigma-2-0mm-3D_glszm_SmallAreaHighGrayLevelEmphasis (*x* ± s). Three IBD and two TA predictive factors were simultaneously included in the multivariate logistic regression analysis, and the combined prediction model was established: (Y-Combine) = -12.33 + 30.76 × log-sigma-20mm-3D_glszm_SmallAreaEmphasis (*x* ± s) - 0.03 × log-sigma-2-0mm-3D_glszm_SmallAreaHighGrayLevelEmphasis (*x* ± s) + 3.31 × intratumoral hemorrhage (present = 0, absent = 1). The three prediction models established in this study could accurately predict OGCTs and OTCA–FTCA (*P* < 0.05). The results of the DeLong test showed that the efficacies of Y-IBD, Y-TA, and Y-Combine were not significantly different (*P* > 0.05; **Figures 4**, **5** and **Tables 5**, **6**).

**Table 5:** Multivariate logistic regression and receiver operating characteristic curve analysis for the overall IBD, overall TA, and combined IBD with TA models.

Features	Multivariate logistic regression analysis				Receiver operating characteristic analysis		
	*B*	*P*-value	Odds ratio	95% CI	AUC	Specificity	Sensitivity
Overall IBD							
Mean ADC (10^3^ s/mm^2^)	6.67	0.015	0.001	0.000 to 0.232	0.685	71.43	65.62
Presence of intratumoral hemorrhage	-4.63	0.020	0.012	0.001 to 0.284	0.815	78.57	84.37
Enhancement degree (solid)	4.67	0.004	102.596	2.055 to 5,121.212	0.683	42.86	93.75
Pre model					0.935	85.71	93.75
Overall TA							
Log-sigma-20mm-3D_glszm_SmallAreaEmphasis	33.18	0.009	3.91	6.540 to 0.0002	0.885	85.71	84.37
Log-sigma-20mm-3D_glszm_SmallAreaHighGrayLevelEmphasis	-0.03	0.036	1.032	1.002 to 1.062	0.795	100.00	71.87
Pre model					0.944	92.86	93.75
Combined IBD and TA							
Presence of intratumoral hemorrhage	3.31	0.030	0.037	0.002 to 0.721	0.815	78.57	84.37
Log-sigma-20mm-3D_glszm_SmallAreaEmphasis	30.76	0.024	4.40	1.089 to 0.018	0.885	85.71	84.37
Log-sigma-20mm-3D_glszm_SmallAreaHighGrayLevelEmphasis	-0.03	0.047	1.034	1.000 to 1.068	0.795	100.00	71.87
Combined model					0.969	92.86	96.87

IBD, imaging-based diagnosis; TA, texture analysis; Pre, prediction.

**Table 6:** Receiver operating characteristic analysis of the overall imaging-based diagnosis (IBD), overall texture analysis (TA), and combined IBD with TA models.

Variables	Difference between areas	Standard error	95% confidence interval	*z* statistic	Significance level (*P*)
Overall_IBD~ Overall_TA	0.009	0.04	-0.067 to 0.085	0.23	0.82
Overall_IBD ~ Combine_IBD_and_TA	0.034	0.03	-0.017 to 0.084	1.30	0.19
Overall_TA ~ Combine_IBD_and_TA	0.025	0.02	-0.020 to 0.067	1.09	0.27

## Discussion

OTCA–FTCA and OGCTs are the most common sex cord stromal tumors and have a low incidence relative to other ovarian tumors. The radiological knowledge of those rare ovarian tumors is still limited in the reported literature; furthermore, the imaging findings of the two entities are similar. Herein we performed a retrospective review of the MRI findings of 32 patients with OTCA–FTCA and 14 patients with OGCTs in this study at our single institution within 7 years. To the best of our knowledge, this is the first study to describe the detailed MRI sign and TA characteristic in this samples.

In our study, the clinical characteristics [age (years), size (maximum), size (average), menopausal status, presence of endometrial hyperplasia, and location] were compared, and there were no significant differences between the two tumors, indicating that they have similar clinical characteristics, as shown in **Table 2**. Combined with literature reports, we found the following: (1) The incidence of intratumoral hemorrhage in this group of OGCTs was as high as 76%, which is higher than that reported in the literature (13). Intratumoral hemorrhage mainly manifested as high signal on T1WI and high signal or low signal on T2WI, and the fluid–fluid level due to hemorrhage could be seen in some lesions. In comparison, the incidence of intratumoral hemorrhage in OTCA–FTCA was only 16% (5/32). The multivariate logistic regression analysis found that the presence of intratumoral hemorrhage could help diagnose OGCTs (OR = 0.12, 95% CI: 0.001–0.284), which is consistent with previous reports that intratumoral hemorrhage is a typical feature of these tumors (14); (2) OTCA–FTCA is composed of theca cells, lutein cells, and fibroblasts. This group of tumors is prone to secondary degenerative changes, such as tumor stromal edema and mucinous degeneration, which may lead to high ADC values (1.50 ± 0.32 × 10^3^ mm^2^/s). In contrast, OGCTs are low-grade malignant tumors that histologically show diffuse, island, beam, follicular, and sarcoma-like growth patterns. These patterns often exist mixed, and the relatively tight arrangement results in more restricted water molecule diffusion with lower ADC values (1.27 ± 0.37 × 10^3^ mm^2^/s) than that of OTCA–FTCA. Therefore, the average ADC value was significantly different between the two tumors (*Z* = -1.982, *P* = 0.047) (15). When the ADC value was ≤1.34 × 10^3^ mm^2^/s, its sensitivity for diagnosing OGCTs was 71.34%, and the specificity was 65.62% (AUC = 0.685, 95% CI: 0.532 to 0.814, *P* = 0.048); (3) In our group, 94% (30/32) of the OTCA–FTCA tumors were mildly enhanced, 6% (2/32) were moderately enhanced, and none showed marked enhancement. In comparison, 57% (8/14) of the OGCTs were mildly enhanced, 43% were moderately enhanced, and none showed marked enhancement. There was a significant difference in the degree of enhancement between the two tumor types (OR = 0.89, 95% CI: 0.015–0.527). It is possible that OTCA–FTCA contains fibrous components, resulting in a lower blood supply and lower enhancement than OGCTs. This is also consistent with previous reports that OTCA–FTCA tumors have a low blood supply, resulting in mild enhancement on MRI (16–18); (4) OGCTs are mostly solid or cystic–solid, and it has been reported in the literature that a “honeycomb” or “sponge” cyst is the characteristic imaging manifestation (19). OTCA–FTCA is often prone to secondary cystic transformation when the tumor volume is large. Some scholars have reported that the cystic transformation rate is 76% (19/25) (20), so the tumor often appears as a cystic–solid or cystic mass, which may be preoperatively misdiagnosed as OGCTs or other ovarian tumor. Other scholars have divided these tumors into solid, cystic, and cystic–solid masses according to the degree of the cystic component. Cystic–solid masses are divided into intratumoral cysts and extratumoral cysts according to whether the cysts are located in the tumor. Intratumoral cysts are divided into peripheral, central, and diffuse types according to their location. The study showed that peritumoral cysts are a characteristic MRI sign (21). In our study, the types of cysts were divided into five degrees according to the degree of cystic degeneration (no cyst: 0°, 0–25%: 1°, 25–50%: 2°, 50–75%: 3°, and greater than 75%: 4°), and the forms of cystic transformation were divided into four forms (no cystic transformation, small cyst, large cyst, and mixed). Between the two tumor types, there was no significant difference in the degree of cystic transformation (Fisher = 0.149, *P* = 0.229), but there was a significant difference in the form of cystic transformation (Fisher = 0.006, *P* = 0.008), indicating that OGCTs mainly demonstrated mixed cystic changes, while OTCA–FTCA predominantly exhibited macrocystic changes. In this study, a weak correlation existed between tumor size and the degree of cystic transformation in the OGCTs group (Kendall’s tau-*b* = 0.618, *P* < 0.001), and no correlation was observed in the OTCA–FTCA group (Kendall’s tau-*b* = -0.025, *P* = 0.857). It is inconsistent with related reports (17) and may be caused by the small sample size.

In the multivariate logistic regression analysis, the IBD model established had an AUC of 0.935, and its sensitivity, specificity, and Youden index were 85.71%, 93.75%, and 0.794 (95% CI: 0.822 to 0.987, *P* < 0.0001), respectively, so the significant features, such as the mean ADC value, enhancement degree, and presence of intratumoral hemorrhage, were important predictors to distinguish between OGCTs and OTCA–FTCA.

TA is different from traditional empirical image analysis based on observations with the naked eye. TA can provide a large amount of imaging information that cannot be recognized by the naked eye by quantitatively analyzing the grayscale information of medical images, realizing the conversion from images to data, and constructing labels to describe the details of the lesion features. Thus, this information could be of value in helping clinicians develop reasonable treatment strategies (22). In recent years, TA has been regarded as an effective means to assess tumor heterogeneity. This method can be used to evaluate the gray-level intensity and position of the pixels within an image to derive texture features that provide a measure of intralesional heterogeneity. TA data are easy to obtain, and no additional imaging is required. In addition, TA plays a relatively important role in evaluating clinical curative effects and predicting prognosis. Many researchers have conducted excellent research, especially with radiomics, in predicting the development trends of tumor lesions (23, 24). The TA in the present study is based on the T2WI sequence because conventional T2WI can reveal the rich histopathological characteristics of tumors, for example, by determining the water content, degree of fibrotic change, necrosis, and hemorrhage (15).

As shown in **Table 4**, the univariate analysis demonstrated that six texture features were significantly different between the OGCTs and OTCA–FTCA groups (*P* < 0.05). Among the six features, the log-sigma-2-0-mm-3D_glszm_SmallAreaEmphasis, log-sigma-2-0-mm-3D_glszm_SmallAreaHighGrayLevelEmphasis, and log-sigma-3-0-mm-3D_glcm_InverseVariance were derived from the image transform type of Laplacian of Gaussian. The wavelet-LLH_glcm_MCC, wavelet-HLH_glszm_SmallAreaHighGrayLevelEmphasis, and wavelet-HLL_glszm_LowGrayLevelZoneEmphasis were derived from the image transform type of wavelet. The features belong to the gray level co-occurrence matrix (GLCM), and the gray level size zone matrix can assess the second-order joint probability function and quantify gray level zones in the image (25). A gray level zone is defined as the number of connected voxels that share the same gray level intensity (26). In the multivariate logistic regression analysis with the forward step method, we found that two features from the image transform type of Laplacian of Gaussian—log-sigma-2-0-mm-3D_glszm_SmallAreaEmphasis and Log-sigma-20mm-3D_glszm_SmallAreaHighGrayLevelEmphasis—are independent risk predictors for distinguishing between OGCTs and OTCA–FTCA (*P* < 0.05). The Laplacian operator can highlight areas in the image where the intensity changes rapidly. The log-sigma-2-0-mm-3D_glszm_SmallAreaEmphasis and log-sigma-20mm-3D_glszm_SmallAreaHighGrayLevelEmphasis describe the distribution of small size zones and the proportion of the joint distribution of smaller size zones with higher gray level values, respectively (27). In our study, the log-sigma-2-0-mm-3D_glszm_SmallAreaEmphasis value of OTCA–FTCA was significantly lower than that of OGCTs, which means that smaller size zones and fine textures were observed in the solid lesions of OTCA–FTCA composed of similar theca cells, lutein cells, and fibroblasts (18). OTCA–FTCA also had significantly less intratumoral hemorrhage than OGCTs in the present study (*P* < 0.05). For the log-sigma-20mm-3D_glszm_SmallAreaHighGrayLevelEmphasis value, the OGCTs obviously contained a greater proportion of the joint distribution of smaller size zones with higher gray level values on T2WI scans than OTCA–FTCA (*P* < 0.05). The log-sigma-20mm-3D_glszm_SmallAreaHighGrayLevelEmphasis is a quantitative index used to compensate for the shortage of MRI findings on T2WI based on solid or cystic components that can be compared. Then, the TA-based predictive model was obtained and had a diagnostic performance/AUC, specificity, and sensitivity of 0.944, 92.86%, and 93.75%, respectively (*P* < 0.05).

The AUC of the IBD and TA combined prediction model to distinguish between OGCTs and OTCA–FTCA was 0.969. When compared with MRI features or TA parameters alone, the combined model showed no significant difference, even though the sensitivity and specificity of the combination were improved to some extent. Therefore, it is believed that the diagnostic performance of the combination model was similar to that of MRI-IBD or T2WI-TA features alone. Furthermore, the diagnostic performance of T2WI-TA parameters was similar to that of MRI-IBD features in helping to distinguish between OGCTs and OTCA–FTCA, which may be less strongly associated with the sample size. However, TA can provide another method to identify OGCTs and OTCA–FTCA.

The present study has several limitations. First of these is the limited study sample size (14 patients with OGCTs and 32 patients with OTCA–FTCA) due to the low incidence of these tumors relative to other ovarian tumors. It might have influence on the final results, such as the rigor of ROC curve analysis. Second, there was an inherent selection bias because the retrospective study was conducted in one institution. We urge the clarification of the imaging findings in larger population-based studies. Third, the ROIs of the ADC and TA were performed manually by radiologists based on individual habits, which may also have influence on the final results. In addition, we did not use MRI images other than T2-weighted images for TA in the present study.

In summary, compared with OTCA–FTCA, OGCTs more commonly exhibit intratumoral hemorrhage, mixed cystic degeneration, moderate enhancement, and low ADC values. Particularly, intratumoral hemorrhage may be a common and characteristic MR finding of OGCTs. When it is difficult to distinguish between OGCTs and OTCA–FTCA, TA described here may serve as a supplementary means, although this will require further large sample size validation before widespread implementation in clinical practice.

## Data Availability Statement

The raw data supporting the conclusions of this article will be made available by the authors, without undue reservation.

## Ethics Statement

The studies involving human participants were reviewed and approved by the ethics committee of the First Affiliated Hospital of USTC, Division of Life Sciences and Medicine, University of Science and Technology of China. Written informed consent to participate in this study was provided by the participants’ legal guardian/next of kin.

## Author Contributions

N-YL, J-ND, and C-BW contributed to conception and design. N-YL organized the database, performed the statistical analysis, and wrote the first draft of the manuscript. BS, P-PW, Y-LC, C-BW, and YC contributed to the collection and arrangement of data. Y-LC, Y-QG and P-PW contributed to data analysis. BS, P-PW, and C-BW wrote sections of the manuscript. All authors contributed to manuscript revision and read and approved the submitted version.

## Funding

This work was supported by the Anhui Provincial Natural Science Foundation for Youths, China (1908085QH364).

## Conflict of Interest

The authors declare that the research was conducted in the absence of any commercial or financial relationships that could be construed as a potential conflict of interest.

## Publisher’s Note

All claims expressed in this article are solely those of the authors and do not necessarily represent those of their affiliated organizations, or those of the publisher, the editors and the reviewers. Any product that may be evaluated in this article, or claim that may be made by its manufacturer, is not guaranteed or endorsed by the publisher.
